# Hand Contact vs Wheel Acceleration: Differences in Propulsion Phase Detection in Manual Wheelchair Users

**DOI:** 10.3390/s26206655

**Published:** 2026-10-21

**Authors:** Luise Krause, Ramin Fathian, Rainer Abel, Chester Ho, Hossein Rouhani, Roy Müller, Lucas Schreff

**Affiliations:** 1 Center for Musculoskeletal Disorders, Klinikum Bayreuth GmbH, 95445 Bayreuth, Germany; 2 Universitätsklinikum Erlangen, Friedrich-Alexander-Universität Erlangen-Nürnberg, 91054 Erlangen, Germany; 3 Department of Mechanical Engineering, Faculty of Engineering, University of Alberta, Edmonton, AB T6G 1H9, Canada; 4 Department of Medicine, University of Alberta, Edmonton, AB T6G 2R7, Canada; 5 Glenrose Rehabilitation Hospital, Edmonton, AB T5G 0B7, Canada; 6 Bayreuth Center of Sport Science, University of Bayreuth, 95447 Bayreuth, Germany; 7 Movement and Exercise Science, Friedrich Schiller University Jena, 07749 Jena, Germany

**Keywords:** spinal cord injury, inertial measurement unit, motion capturing, activity monitoring, continuous wavelet transform

## Abstract

**Highlights:**

**What are the main findings?**

- Approach 2 (hand-acceleration-based) detects longer propulsion and shorter recovery phases than Approach 1 (wheel acceleration-based), with differences varying by speed conditions.
- Non-propulsive hand–pushrim contact may contribute to the difference between approaches, but this mechanism was not directly verified in this study.

**What are the implications of the main findings?**

- Propulsion phase durations reported by different measurement approaches are not directly comparable, so studies should clearly report which method was used.
- Researchers and clinicians should account for the measurement approach when interpreting or pooling propulsion biomechanics data across studies.

**Abstract:**

(1) Background: Accurate detection of propulsion and recovery phases is essential for assessing wheelchair propulsion biomechanics. Different measurement approaches rely on distinct biomechanical signals and may therefore yield systematically different phase durations. (2) Methods: This study compared propulsion and recovery phase durations obtained from a wheel acceleration-based approach (Approach 1: marker-based motion capture) and a hand-acceleration-based approach (Approach 2: hand-mounted IMU using the continuous wavelet transform, CWT) in 33 manual wheelchair users with neurological conditions across three propulsion speeds (slow, preferred, fast). (3) Results: Approach 2 detected longer propulsion phases (bias: +0.13 s, limits of agreement (LoA) ± 0.30 s) and shorter recovery phases (bias: −0.13 s, LoA ± 0.30 s) than Approach 1. Both biases varied with speed conditions and diminished as propulsion speed increased. The later end event detected by Approach 2 is compatible with the hypothesis that hand–pushrim contact can continue after wheel acceleration changes, potentially including non-propulsive contact late in the push phase. However, this mechanism was not directly verified: no independent pushrim-force or hand-contact reference was collected. (4) Conclusions: The two approaches operationalize propulsion phase timing using different signals and should not be assumed to identify identical events. The observed differences limit direct comparison of propulsion parameters across studies unless the measurement approach is reported.

## 1. Introduction

It is estimated that there are around 132 million people worldwide who need a wheelchair, but the number of unreported cases might be significantly higher. Furthermore, demographic changes and population aging are expected to increase the number of people relying on wheelchairs in the coming decades [1,2]. Among wheelchair users, manual wheelchairs are the most common mode of mobility [3].

Manual wheelchair use places considerable mechanical demands on the upper extremities. A systematic review and meta-analysis [4] reported a pooled prevalence of musculoskeletal pain of 50% among wheelchair users, with shoulder pain as the most common pain syndrome (pooled prevalence 44%). Other commonly affected sites included the neck, elbow, wrist, and hand [4]. These mechanical demands are further increased by suboptimal seating position or propulsion technique [5,6,7,8,9]. Timely analysis of propulsion technique and seating position is therefore important for identifying and addressing factors that contribute to upper-limb overuse injury [10,11].

**Figure 1:**
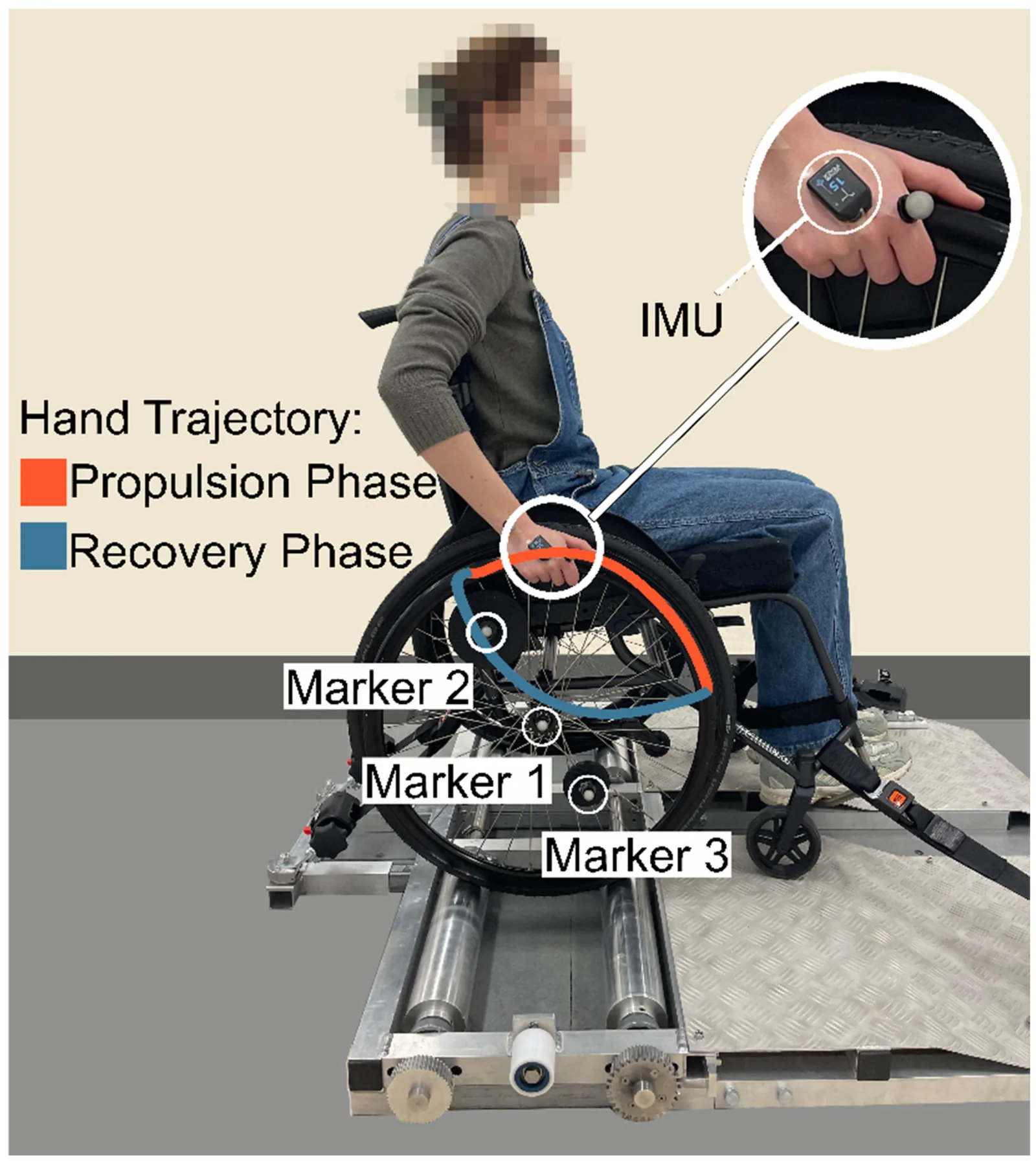
The measurement setup: Participants propelled their own wheelchairs while the wheelchair was secured on a test stand using straps. Wheel motion was recorded using three reflective markers attached to the wheel, and hand motion was recorded using an IMU sensor placed on the right hand. The marker placed on the first knuckle (2nd MCP joint) of the participant’s right hand was not used in this study.

Manual wheelchair propulsion is a cyclic movement in which users apply force to the pushrim to propel the wheelchair (hand–pushrim interaction). Each cycle is commonly divided into a propulsion phase, during which the hands drive the wheel forward, and a recovery phase, during which the hands return to the starting position without driving the wheel (Figure 1). Accurate detection of these phases is fundamental for assessing spatio-temporal parameters in manual wheelchair propulsion. However, the exact start and end of each phase depend on the detection method, and no standardized approach exists. Published methods derive the phase transitions from different signals, including wheel acceleration [12,13,14], pushrim force [15,16], and hand movement or acceleration [9,17,18]. Because each signal represents a different biomechanical event, these methods may yield different phase durations for the same propulsion cycle.

One approach is to define propulsion phases based on changes in the wheel acceleration. Under laboratory conditions, this can be achieved using marker-based camera systems with reflective markers attached to the wheel [14], while wheel-mounted inertial measurement units (IMUs) offer a portable and cost-effective alternative [12]. In a recent study, Karner et al. [13] compared both systems to validate their use in wheelchair propulsion detection on a test stand. They reported high agreement between spatio-temporal parameters derived from the IMU system and those from the camera-based system. However, while both systems measure wheel acceleration reliably, the period of positive wheel acceleration is not necessarily identical to the period of hand–pushrim contact. Hand contact may extend beyond positive wheel acceleration [15]. Therefore, phase detection based on wheel acceleration may underestimate the contact phase, regardless of the measurement system used.

As an alternative approach to propulsion phase detection, recent studies attempted to determine hand-event instants [9] and classify propulsion patterns [19] using hand-mounted IMUs and analyzing hand acceleration using continuous wavelet transform (CWT). In a study by Fathian et al. [9], the IMU-based algorithm was compared with the SmartWheel (force sensors embedded into the pushrim [16]) and good agreement was observed between the detected propulsion events.

The differences in propulsion phase duration obtained by different methodological approaches may be further influenced by propulsion velocity, propulsion techniques, user demographics, or upper-limb sensorimotor impairment. Investigating such differences can be used not only to choose the propulsion detection approach but also to characterize the hand–pushrim interaction in various user and propulsion conditions. Therefore, the present study aims to investigate the relationship between hand-acceleration-based and wheel acceleration-based measurements of push and recovery phase durations in manual wheelchair users with neurological conditions at three different speeds (preferred, slow, and fast). By comparing these measurement approaches across speeds, this study seeks to provide guidance for future research on the determination of propulsion phases and to improve the interpretation and comparability of studies using different methodological approaches.

## 2. Materials and Methods

### 2.1. Participants

A total of 33 participants were recruited in the Center for Musculoskeletal Disorders, Klinikum Bayreuth GmbH, Germany (Table 1). Inclusion criteria were as follows: age 18–65 years, individuals with paraplegia or tetraplegia who use manual wheelchairs, and the ability to push the wheelchair at least 100 m. Participants with complaints commonly associated with long-term wheelchair use were not excluded. The only exclusion criterion was the use of an electric add-on drive (power-assist) on the wheelchair. All participants provided written informed consent to participate in the study. The investigation was approved by the ethics review board of the Friedrich-Alexander University Erlangen-Nürnberg, Germany (23-20-B; 14 February 2023) and was in accordance with the Declaration of Helsinki.

### 2.2. Set-Up and Measurement

The measurements took place in the gait and movement laboratory at Klinikum Bayreuth GmbH, Germany. A marker-based motion capture system (VICON, Oxford, UK) including 10 infrared cameras (VICON Vero v2.2; 200 Hz; 2048 by 1088 pixels) was used to monitor participant movement. Three markers were placed on the right wheelchair wheel: one in the middle (marker 1) and two on the spokes (markers 2 and 3; see Figure 1). A WaveTrack IMU sensor (Cometa, Bareggio, Italy) was placed on the skin over the right third metacarpal bone, with the acceleration range set to 8 g. Native IMU data were acquired at 284 Hz for the first three participants and 142 Hz for the remaining participants. Vicon Motion Systems Ltd. (Oxford, UK) and Nexus 2.16 synchronized the IMU and marker data and resampled the IMU stream from its native rate to the system global analog sampling rate of 2000 Hz. Subsequent IMU processing used this synchronized, resampled stream; no additional resampling was applied by the analysis pipeline.

Upon arrival, participants were informed about the study procedure and provided written informed consent. Demographic and clinical characteristics were then recorded (Table 1). Subsequently, markers and IMUs were attached. Three researchers were involved in the attachment across all measurements. At each measurement, at least one additional researcher cross-checked the positioning of all markers and sensors. All participants propelled their own adapted wheelchairs on a test stand (Figure 1). After the wheelchair was placed on the test stand, it was secured with straps to provide additional safety for the participants. Participants familiarized themselves with propelling in place on the test stand for as long as they needed, and data collection started once they indicated that they felt ready.

Participants were instructed verbally to propel their wheelchairs in three conditions, in this order: at their preferred speed, at a slower speed than preferred, and at a faster speed than preferred. Speeds were self-selected in all conditions, and no target speeds were prescribed. The preferred speed was not determined by a separate measurement (e.g., cost of transport). Speeds were therefore individual to each participant.

Each speed condition was recorded for 15 consecutive push cycles, regardless of the time required. Participants rested between conditions for as long as they needed before continuing. To exclude the transition phase at the beginning and end of each trial, only 10 cycles in the middle of the 15 cycles were considered for data analysis.

### 2.3. Data Processing

Approach 1 (wheel acceleration measurement): In the wheel acceleration-based approach, the start and end times of the cycle phases were determined from the angular acceleration of the wheels, following the procedure described by Karner et al. [13]. Gaps in the marker trajectories, caused by occlusion of marker 2 by the hand during propulsion, were filled using a Vicon Rigid Body Fill, exploiting the known rigid-body geometry of markers 1, 2, and 3 mounted on the wheel. Marker 3 served as a reference to reconstruct the position of marker 2 when occluded. The gap-filled trajectories were then processed in Python (version 3.13.4), within the PyCharm development environment (version 2021.3, JetBrains, Prague, Czech Republic) by applying a second-order Butterworth low-pass filter with a 6 Hz cutoff frequency [20]. Angular acceleration was derived from wheel markers 1 and 2 (Figure 1). Zero crossings of the angular acceleration signal (i.e., transitions from positive to negative values or vice versa) were used to identify the start and end times of the propulsion phase.

Approach 2 (hand-acceleration measurement): Hand-event timing was estimated using the CWT-based method described by Fathian et al. [9]. The resultant acceleration was calculated as the Euclidean norm of the triaxial IMU acceleration and low-pass filtered with a sixth-order Butterworth filter (cutoff frequency: 10 Hz). Prior to CWT analysis, a trend removal step estimated and subtracted the slowly varying baseline from the resultant-acceleration signal. This step requires a sufficiently stationary signal segment at the beginning and end of each trial. As trials were fixed at 15 push cycles with no mandatory rest period, insufficient resting signal at very low propulsion speeds could result in unreliable baseline estimation, causing event detection to fail; trials affected in this way were excluded from the analysis. The filtered signal was divided into M splits using an overlapping sliding window. CWT decomposition used a Morlet mother wavelet and scales from 1 to the maximum scaling factor (S_max_); Figure 3 of Fathian et al. [9] illustrates scales up to 500. Scale-independent energy (E_s_), calculated from the CWT coefficients, was used to identify two dominant scales: the low-frequency cycle scale (µ_c_) and the higher-frequency event scale (µ_e_), with µ_e_ < µ_c_. The cadence-adaptation mechanism cross-correlated the energy profile for each split with the preceding estimate to update the target scales between windows. CWT coefficients at the adapted scales defined temporal regions of interest. Within these regions, contact and release event times were assigned to the locations of the highest unfiltered resultant-acceleration values. Approach 2 therefore provides acceleration-derived, contact-related event estimates rather than a direct measurement of hand–pushrim contact or applied force.

### 2.4. Statistical Analysis

Statistical analyses were conducted using Python (version 3.13.4), within the PyCharm development environment (version 2021.3, JetBrains, Prague, Czech Republic). The propulsion parameters, including propulsion phase duration, recovery phase duration, as well as the time points t_start_ and t_end_ obtained by Approaches 1 and 2, were calculated and compared together. In this study, the term propulsion phase is used as a general descriptor; however, its definition depended on the underlying measurement signal. For Approach 1, the propulsion phase corresponds to the push phase, i.e., when the applied hand torque exceeds the resistive torque. For Approach 2, the propulsion phase corresponds to the contact phase (period of hand–pushrim contact). Accordingly, t_start_ and t_end_ denote the beginning and end of the propulsion phase as defined by each respective approach. Data from three propulsion speeds (slow, preferred, and fast) were analyzed. Only propulsion cycles with valid detections from both systems were included. Descriptive statistics are reported as mean and standard deviation. The correspondence between the two measurement approaches was further characterized using Spearman’s correlation coefficient [21].

Additionally, Bland–Altman plots were used to visualize differences between Approaches 1 and 2. Bland–Altman plots [22,23] were generated for each parameter, displaying the mean of both approaches against their difference, together with the bias (mean difference between systems) and the 95% limits of agreement (LoA). For event detection analysis, t_start_ and t_end_ were analyzed separately. Event timing was normalized across the ten analyzed propulsion cycles, where the first and tenth marker-detected events corresponded to 0% and 100%, respectively. Thus, normalization was referenced to both approaches, and IMU events were mapped onto the same temporal scale. For all other propulsion parameters, regression lines were plotted in Bland–Altman plots to visualize trends in the differences between the two approaches.

To assess whether the differences between the marker-based and IMU-based measurements were influenced by propulsion speed, a repeated-measures ANCOVA was performed, with measurement method as the within-subject factor and actual propulsion speed as a continuous covariate. An alpha level of 0.05 was used for all statistical tests.

## 3. Results

**Figure 2:**
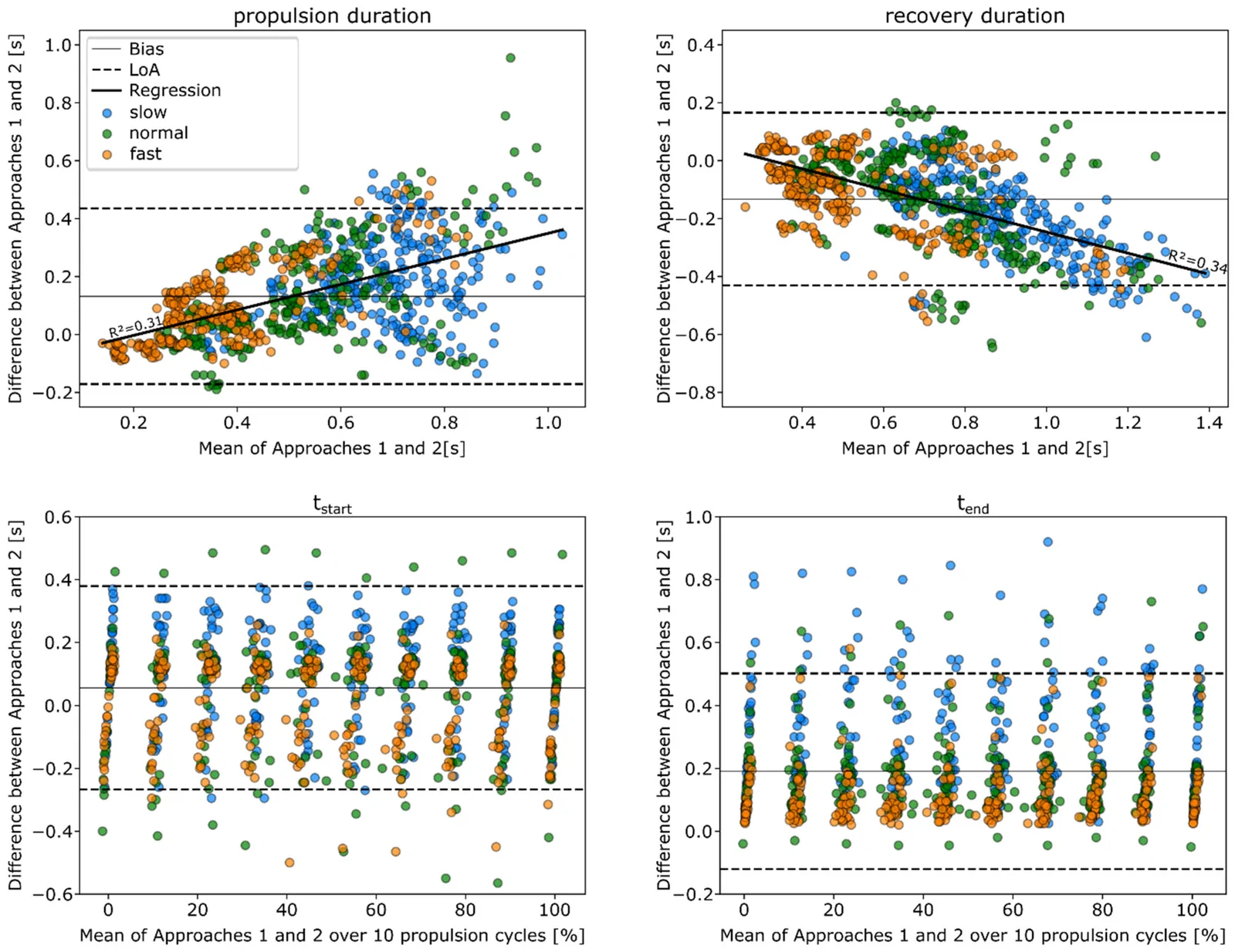
Bland–Altman plots comparing wheel acceleration-based (Approach 1) and hand-acceleration-based (Approach 2) measurements for wheelchair propulsion parameters: propulsion duration, recovery duration, t_start_, and t_end_. Positive values on the vertical axis indicate longer phase durations or later start and end times for Approach 2 compared to Approach 1. Data points are color-coded by propulsion velocity (blue = slow, green = preferred, orange = fast). The solid narrow line represents the mean bias, dashed horizontal lines indicate the limits of agreement (±1.96 SD), and the solid broad line represents the regression line.

The results of our comparison between both measurement approaches are shown in Table 2 and visualized in Figure 2. For three participants, slow-velocity trials were excluded from the analysis due to failed event detection in Approach 2. Across the remaining trials, t_start_ events were detected by Approach 2 in 947 of 960 cases (98.6%), and t_end_ events were detected in 958 of 960 cases (99.8%).

Mean propulsion speeds (detected by the marker system) across conditions were 0.86 ± 0.45 m/s (preferred), 0.43 ± 0.23 m/s (slow), and 1.59 ± 0.90 m/s (fast). Propulsion duration derived from the IMU system (Approach 2) was systematically longer compared to the wheel acceleration-based system (Approach 1), with an overall bias of 0.13 s and LoAs of ±0.30 s (r = 0.8323, *p* < 0.001). This difference was velocity-dependent (R^2^ = 0.31; Figure 2), with biases of 0.19 s for slow, 0.12 s for preferred, and 0.09 s for fast propulsion. Repeated-measures ANCOVA confirmed a significant effect of propulsion speed on the difference between methods for propulsion duration (F(1, 945) = 37.0, *p* < 0.001). Recovery duration derived from the IMU system was systematically shorter, with an overall bias of −0.13 s and LoAs of ±0.30 s (r = 0.8695, *p* < 0.001). The difference was −0.19 s at slow, −0.12 s at preferred, and −0.10 s at fast propulsion, indicating that inter-system differences diminished with increasing propulsion speed (R^2^ = 0.34, Figure 2). This speed-dependent difference was likewise confirmed by repeated-measures ANCOVA for recovery duration (F(1, 945) = 36.9, *p* < 0.001).

t_start_ and t_end_ of the propulsion phase derived from Approach 2 were detected later compared to Approach 1. For t_start_, the overall bias was 0.06 s with LoAs of ±0.32 s. Across velocity conditions, the difference increased with slower propulsion (slow: 0.12 s; preferred: 0.05 s; fast: 0.01 s). Repeated-measures ANCOVA confirmed a significant speed effect on the difference for t_start_ (F(1, 946) = 182.3, *p* < 0.001). t_end_ showed a similar pattern, with an overall bias of 0.19 s and LoA of ±0.31 s. The difference decreased progressively from slow (0.31 s) to preferred (0.17 s) and fast conditions (0.11 s), indicating better correspondence between systems at higher propulsion velocities. A significant speed effect was also confirmed for t_end_ (F(1, 957) = 572.2, *p* < 0.001).

Propulsion frequency did not differ significantly between the two systems (marker-based: 0.66 ± 0.13 Hz, 0.91 ± 0.26 Hz, and 1.27 ± 0.39 Hz for slow, preferred, and fast conditions, respectively; IMU-based: 0.66 ± 0.14 Hz, 0.92 ± 0.27 Hz, and 1.28 ± 0.34 Hz, respectively), and no significant effect of propulsion speed on this correspondence was found (F(1, 940) = 0.075, *p* = 0.784).

## 4. Discussion

The present study addresses a methodological gap in characterization of manual wheelchair propulsion biomechanics: whether approaches to determine propulsion phases based on different biomechanical principles (e.g., wheel acceleration or hand-acceleration change) lead to systematically different outcomes. Our main finding was that these approaches influenced the duration of the detected propulsion phases, and that these differences varied with propulsion speed.

Approach 2 yielded a longer propulsion phase and a consequently shorter recovery phase compared to the camera-based system in Approach 1. The differences were smaller at higher propulsion speeds and larger at lower speeds. Both t_start_ and t_end_ events were detected later by Approach 2. For t_start_, the overall difference was 0.06 s. This is a difference between events defined by two measurement signals; because Approach 2 estimates contact-related events from hand acceleration, it should not be interpreted as a direct observation of hand contact or as evidence that contact began after pushing. Although hand–pushrim contact would biomechanically be expected to precede force application and the resulting wheel acceleration, that event sequence was not independently measured in this study.

The later t_end_ event from Approach 2 is compatible with continued hand–pushrim contact after the wheel acceleration-based criterion marks the end of the push phase. Non-propulsive pushrim contact late in the stroke has been described previously [15], making it a plausible explanation for the observed timing difference. However, this study included no independent pushrim-force measurement or hand-contact reference, so the timing offset does not demonstrate that Approach 2 detected non-propulsive contact; the mechanism remains a hypothesis. It may be more relevant at lower propulsion speeds, when applied forces may be insufficient to overcome the resistance of the roller ergometer, but this interpretation requires direct validation. Instrumented wheels measure pushrim force and can identify applied force even when it does not produce positive net wheel acceleration. In a previous comparison with an instrumented wheel system (SmartWheel [16]), Fathian et al. [9] reported smaller differences in phase duration (mean error ± SD: 0.01 ± 0.06 s for push duration and −0.02 ± 0.08 s for recovery duration) and also observed later t_start_ and t_end_ events with the hand-acceleration approach.

This study has several limitations. First, propulsion speeds were self-selected rather than standardized across participants. Absolute speeds therefore differed between individuals, which may have influenced the detected phase durations. Second, measurements were conducted on a stationary test stand without external loading or resistance, which does not fully replicate the mechanical demands of overground propulsion. Third, detailed neurological characteristics (e.g., ASIA impairment scale, neurological level) and several wheelchair configuration parameters (e.g., seat height, rear axle position, camber) were not recorded, although these factors may influence hand–pushrim interaction and propulsion biomechanics. Finally, in the absence of an independent criterion measure such as pushrim force, neither approach can be considered universally more accurate.

From a practical perspective, force-based approaches provide a direct measure of the applied pushrim force and are therefore well suited to determine propulsion phase durations. However, the availability of instrumented wheels in clinical and laboratory settings is often limited. As a result, hand and wheel acceleration-based approaches represent practical alternatives, since they can be implemented using hand- or wheel-mounted IMUs [9,12,13]. Given the speed-dependent differences between both approaches, pooling data or applying a universal correction factor across methods is discouraged without further validation, and future studies should clearly report the detection method and speed range used. The choice of approach should depend on the intended application. Approach 1 enables propulsion speed to be determined and an estimation of the moment at which the hand actually transmits propulsive force to the pushrim. However, its application in the field may be affected by additional accelerations caused by terrain conditions or by upper body and arm movements during the recovery phase, which could make phase detection more difficult [12,24]. In contrast, Approach 2 is more readily applicable to overground measurement and provides additional hand-related data, such as hand trajectories [19], that are inaccessible through wheel-based sensing.

Combining hand- and wheel-mounted IMUs may be a promising approach for future studies, as it could improve the robustness of propulsion phase identification in real-world conditions. Furthermore, this approach would allow both wheel-related parameters and hand-related kinematics to be assessed. Therefore, future research should investigate whether such combined sensor approaches can enhance propulsion phase detection and provide a more comprehensive characterization of wheelchair propulsion in field-based settings.

## 5. Patents

R.F., C.H., and H.R. hold a pending patent related to the IMU-based wheelchair propulsion monitoring method described in this study.

## Figures and Tables

**Table 1:** Participant characteristics (paraplegia: para, tetraplegia: tetra).

	Mean ± SD
Participants [n]	33
Injury level [n]	28 para, 5 tetra
Sex [n]	7 W, 26 M
Age [years]	47.8 ± 14.6
Body mass [kg]	79.3 ± 19.3
Arm length [cm]	54.5 ± 4.5
Wheel diameter [in]	23.7 ± 0.9
Time in wheelchair [years]	16.3 ± 13.6

**Table 2:** Comparison of the propulsion and recovery duration detected by both measurement approaches: Approach 1 based on wheel acceleration and Approach 2 based on hand acceleration. Mean values ± standard deviations (SD) are presented, along with bias ± limits of agreement (LoA) and Spearman’s correlation coefficients (r).

	Mean ± SD (Approach 1)	Mean ± SD (Approach 2)	Bias ± LoA	r
Propulsion duration [s] all conditions slow preferred fast	0.44 ± 0.17 0.59 ± 0.13 0.44 ± 0.14 0.31 ± 0.10	0.57 ± 0.25 0.78 ± 0.16 0.56 ± 0.23 0.40 ± 0.19	0.13 ± 0.30 0.19 ± 0.29 0.12 ± 0.33 0.09 ± 0.25	0.8323 0.4739 0.7239 0.7127
Recovery duration [s] all conditions slow preferred fast	0.75 ± 0.30 0.98 ± 0.24 0.74 ± 0.26 0.57 ± 0.23	0.62 ± 0.21 0.79 ± 0.14 0.62 ± 0.18 0.47 ± 0.17	−0.13 ± 0.30 −0.19 ± 0.28 −0.12 ± 0.32 −0.10 ± 0.25	0.8695 0.8624 0.7947 0.7270

## Data Availability

The raw data supporting the conclusions of this article will be made available by the authors on request. The data are not publicly available due to privacy reasons.

## Abbreviations

The following abbreviations are used in this manuscript:

Bias	Mean difference between systems
CWT	Continuous wavelet transform
E_s_	Scale-independent energy
IMU	Inertial measurement unit
LoA	Limits of agreement
MCP	Metacarpo-phalangeal
μ_c_	Cycle scale
μ_e_	Event scale
S_max_	maximum scaling factor
t_start_	Start of propulsion phase
t_end_	End of propulsion phase
